# Anaplastic Thyroid Cancer: The Addition of Systemic Chemotherapy to Radiotherapy Led to an Observed Improvement in Survival—A Single Centre Experience and Review of the Literature

**DOI:** 10.1155/2014/674583

**Published:** 2014-02-17

**Authors:** Natalie M. Lowe, Sean Loughran, Nicholas J. Slevin, Beng K. Yap

**Affiliations:** ^1^Head and Neck Surgery, The Christie NHS Foundation Trust, Wilmslow Road, Manchester M20 4BX, UK; ^2^Head and Neck Surgery, Manchester Royal Infirmary, Oxford Road, Manchester M13 9WL, UK; ^3^Head and Neck Oncology, The Christie NHS Foundation Trust, Wilmslow Road, Manchester M20 4BX, UK

## Abstract

*Introduction*. Anaplastic thyroid carcinoma (ATC) is rare yet accounts for up to 50% of all thyroid cancer deaths. This study reviews outcomes of patients with confirmed ATC referred to a tertiary oncology centre plus reviews the literature to explore how poor outcomes may be improved. *Materials and Methods*. The management and outcomes of 20 patients with ATC were reviewed. *Results*. Median age at diagnosis was 69.5 years. 19 patients died due to ATC, 40% of whom died from asphyxiation. Median survival for all cases was 59 days. Patients who had previous surgery prior to other treatment modalities had a longer median survival overall compared to those who had not had previous surgery (142 days compared to 59 days) and produced the one long-term survivor. Chemotherapy followed by radiotherapy (without previous surgery) was associated with longer median survival (220 days). Palliative radiotherapy alone did not decrease the rate of death by asphyxiation when compared to other single modality treatments. *Conclusion*. Multimodality treatment including surgery when feasible remains the best strategy to improve survival and prevent death from asphyxiation in the management of ATC. The addition of chemotherapy to our institutional protocol led to improved survival but prognosis remains very poor.

## 1. Introduction

Anaplastic thyroid carcinoma (ATC) is rare, representing only 1-2% of all thyroid cancers, but nevertheless accounts for up to 50% of all thyroid cancer deaths [1–6]. Survival time is generally short with one-year survival rates being reported at 10–20% [6–10]. Long-term survival is rare and can lead to doubts about diagnosis [3, 6–8]. With the small number of patients diagnosed with ATC and the rapid progression of the disease, there is little consensus about the best treatment approach for newly diagnosed patients or in which order the treatment modalities should be offered [6, 11, 12]. The American Thyroid Association has recently published guidelines for the management of patients with ATC, [13], though despite an extensive review of the literature to produce these guidelines, the quality of evidence available to produce the 65 recommendations is reported by the authors as being either low or moderate only. This paper aims to discuss our experience and outcomes of a series of 20 consecutive patients referred to a tertiary oncology unit with a diagnosis of ATC. Patterns noticed within the outcomes will be compared to the current literature and recommendations. How dismal outcomes may be improved will also be considered.

## 2. Findings at Our Institution

### 2.1. Materials and Methods

We searched for all cases of ATC in our institutional prospective thyroid cancer database from 2004 to 2011. Patients were only included into the study when they had histologically confirmed ATC via core biopsy or surgical specimen based on WHO classification. All patients had already been deemed not suitable for surgery or further surgery by their referring thyroid surgeons. Our institutional practice was to offer radiotherapy only in 2004–2007. Due to the disappointing outcome, we introduced chemotherapy in our institutional protocol since 2008 to be followed by radiotherapy if patient's performance status permitted. The intended number of cycles of chemotherapy used in our institution was 6. The chemotherapy regimens used were cisplatin 60 mg/m^2^ or carboplatin (area under the curve = 5) in patients with poor renal function and doxorubicin 60 mg/m^2^ or taxotere 75 mg/m^2^ on a 3-weekly basis. Patients who had both chemotherapy and radiotherapy always started with chemotherapy followed by radiotherapy in this series; concurrent chemoradiotherapy treatment was not given. Formal statistical analysis to detect statistically significance differences was not performed due to the small number and wide variation of patient and treatment characteristics.

### 2.2. Results

20 out of 1030 patients in the thyroid cancer database met ATC criteria and hence were included in the final review. The ratio of female to male subjects was 9 female to 11 male (ratio 1 : 1.2) with the median age at diagnosis being 69.5 years (range 56.3–80.56 years).

### 2.3. Pattern of Spread at Presentation

12 patients (60%) were found to have metastatic disease at presentation. All of these were found to have lung metastases; of these 12, two also had bone metastases, one had adrenal metastases, one had brain metastases, and one had hepatic metastases.

### 2.4. Mode of Death

19 patients (95%) died due to the anaplastic thyroid carcinoma or as a consequence of its complications, eight (40%) of whom died specifically from asphyxiation. The mode of death was not known in one of the patients. Two patients had tracheostomies, one of whom died from asphyxiation.

### 2.5. Survival

Median survival for all cases was 59 days (ranging from 32 to 350 days plus one long-term survival (eight years plus)) (calculated from date first seen at our centre rather than the referring institution). One patient is still alive, with no signs of recurrence greater than eight years since diagnosis. This is the only person to have survived a year or more (1 year survival rate = 5%).  Table 1 shows the number of patients having each treatment regimen and their median survival plus the percentage of each treatment group that died from asphyxiation.

The median survival of all treatment groups of patients who had had surgery prior to referral was found to be 142 days (range 32 days–eight years+) compared to a median survival of 59 days (range 32–350 days) for those who had not had surgery. Chemotherapy followed by radiotherapy however produced the longest median survival out of all treatment modalities at 220 days (range 52–350 days).

### 2.6. Performance Status

On analysis of the World Health Organisation (WHO) performance scores for patients receiving different treatment modalities, our series showed a good spread of WHO scores. Hence, outcomes to different treatment modalities could not be specifically attributed to this.

## 3. Comparison with the Current Literature

### 3.1. Survival

In line with current consensus, we found ATC to usually be a disease of the elderly (median age at diagnosis: 69.5 years (range 56.3–80.56 years)). Our series showed a slight male preponderance (1 : 1.2) which is reflected by some studies [6], but many suggest a greater female preponderance [7, 8, 12, 14, 15]. As reflected in previous studies, the prognosis and one-year survival rates for these patients with ATC were poor [6, 7, 9, 10]. Our series demonstrated the median survival to be 59 days and a one-year survival rate of 5% from time of first consultation at our institution. The one-year survival is lower than other previously reported studies (10–20%) [6, 7, 9, 10]. However, comparison with older studies is complicated by uncertainty of histological diagnosis in some studies. Some histopathology specimens that might have historically been labelled as anaplastic would actually now be labelled as other types of carcinoma using today's criteria, which may be either less aggressive or have a better response to the nonsurgical treatment modalities such as non-Hodgkin's lymphoma [3, 6–8]. Indeed, the US national database from 1985 to 1995 reports a 5-year survival as high as 55% in certain groups of patients (aged <46 years old); however, this also reports that there had been no independent pathology review [14]. The current definition and histological findings of ATC as stated by the WHO are shown as follows [16].

*WHO Classification of Tumours: Undifferentiated/Anaplastic Thyroid Carcinoma [16]:*

*Undifferentiated Thyroid Carcinomas (UTC) are highly malignant tumours that histologically appear wholly or partially composed of undifferentiated cells that exhibit immunohistochemical or ultra-structural features indicative of epithelial differentiation. Histologically they are composed of a mixture of spindle cells, pleomorphic giant cells and epithelial cells. There is considerable variation in both the percentage and distribution of these cellular elements from case to case.*

### 3.2. Surgery

Our study showed that the seven patients who had previous surgery had a longer median survival (142 days; range 38 days–>8 years) as compared to the 13 patients who had not had previous surgery (59 days; range 32–350 days). The only long-term survivor in our series also had surgery prior to radiotherapy, a treatment strategy that was also shown to significantly increase survival by Kobayashi et al. [17]. Haigh et al. [12] performed an analysis of 33 patients reporting that multivariate analysis of the factors associated with significant survival differences revealed that potentially curative surgery was the only discriminating variable that retained a significant association with prolonged survival. Our data would support this view that surgery is an important modality of treatment in the management of ATC, and when feasible, we would recommend it as part of multimodality treatment. Obviously, there is a potential bias towards surgery being done in fitter patients with less advanced local disease; however, in our series there was a good spread of performance statuses across both groups. Contrary to expectations, the median performance status was lower in the nonsurgical group (WHO PS = 0) compared to the surgical group (WHO PS = 1), which also included the patient with the poorest performance status in the whole series (WHO PS = 3).

Further surgery was not offered following referral to our institution as it was deemed not beneficial by the referring thyroid surgeons; therefore, only 35% of the series had any form of surgery as part of their treatment. This may have contributed to the poor survival in our series as compared to other series [6, 7, 9, 10] which may have been more aggressive in offering surgical treatment.

### 3.3. Radiotherapy

Our institutional protocol from 2004 to 2007 was to offer radiotherapy in the postoperative setting and for inoperable cases. The median dose given was 30 Gy (range 24–65 Gy). The median survival in the radiotherapy only group was 59 days (range 42–206 days) from first consultation at our institution. This is similar to the overall median survival for the entire cohort (59 days) and only 27 days greater than no treatment at all. These dismal outcomes lead to the change of our institutional protocol by the addition of chemotherapy since 2008. However, studies often suggest that radiotherapy is worthwhile as it does improve outcome, especially when performed after surgery [6, 8, 9]. This result was reflected in our series in which patients who had received radiotherapy after surgery had a median survival of 176 days (range 4 days–>8 years+) and improved from 38 days from surgery alone.

### 3.4. Chemotherapy

Six patients in our series had chemotherapy, 3 of these were in combination with radiotherapy. The median survival of patients treated with chemotherapy as a single therapy agent was 137 days (range 41–175 days) which was improved from other single agent treatment modalities (surgery 38 days (range 32–238 days) and radiotherapy 58.5 days (range 42–206 days)). Combining chemotherapy with radiotherapy (median dose 45 Gy; range 30 Gy–55 Gy) produced the longest median survival in our series at 220 days (range 52–350 days) even when compared to surgically treated patients, although chemotherapy was not given in combination with surgery in this series. Contrary to expectations, the chemotherapy plus radiotherapy treatment group had the highest median WHO PS in our series (median PS = 1 compared to 0 in radiotherapy and chemotherapy groups), and hence good results could not be solely attributed to this. Although these results are too small to draw definite conclusions from, they do correspond with several other studies that suggest that chemotherapy is of benefit in the management of ATC [10, 12, 18] rather than with some studies which did not find any proven benefit [9, 11, 19]. Our series highlighted the important question of balancing possible modest improvement in survival versus quality of life. We defined 6 cycles of chemotherapy to be the intended course length of chemotherapy. However, 5 out of 6 (83%) patients did not finish their intended course due to poor tolerance and side effects. The median number of cycles of chemotherapy actually received was 3 (range 2–6). In retrospect, the 6 intended courses of chemotherapy may have been overly ambitious.

### 3.5. Asphyxiation

**Figure 1:**
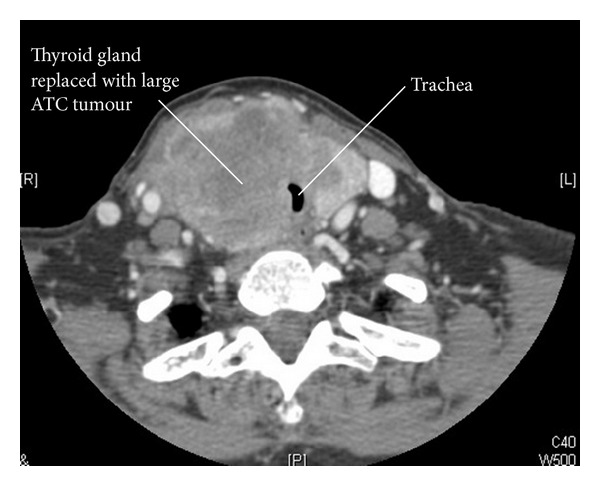
CT scan of patient with ATC causing tracheal compression.

ATC often presents with locally advanced large tumours as shown in the CT scan in Figure 1. Death in ATC patients is often therefore caused by tracheal and oesophageal invasion and obstruction, as well as by consequences of metastatic disease. We found that 8 out of 20 patients (40%) died from asphyxiation. All the 3 patients who received chemotherapy followed by radiotherapy died of asphyxiation in our series. This group had the longest median survival time and hence perhaps allowed time for local tumour growth. As with our practice, several centres offer radiotherapy as palliation to prevent local and compression symptoms. However, our series showed that the number of deaths from asphyxiation was the same for all patients receiving single modality treatments such as radiotherapy (33%), chemotherapy (33%), and surgery (33%) (see Table 1). These results may be borne in mind when only offering palliative dose radiotherapy to prevent local and compression symptoms as is often done [3, 7, 11, 18].

## 4. Discussion: How Can Outcomes Be Improved?

### 4.1. Correct and Swift Diagnosis

In order to improve outcomes for patients with ATC, it is first vital to get a correct and swift diagnosis so that treatment choice can be tailored to their needs. Correct histopathological diagnosis is essential in order to differentiate ATC from other less aggressive and more treatable tumours that can mimic ATC closely. Samples can be obtained from either fine needle aspiration techniques, core biopsy,or open biopsy [13]. ATC is a rapidly progressing disease and hence once formally diagnosed, although clinical assessment of the extent of the local, regional, and distant disease should ideally be obtained, as per the ATA guidelines for the treatment of ATC, these investigations (such as the biopsy of suspected metastatic disease) should not delay the definitive treatment [13]:

*It is vital to get a correct and swift diagnosis of ATC; differentiating it from other less aggressive diagnoses that can mimic closely. Further investigations such as the biopsying of suspected metastatic disease should not delay the prompt initiation of definitive treatment.*

### 4.2. Surgery

A decision should be made initially whether the tumour is resectable or unresectable. Most studies suggest that treatment regimens that include complete resection are associated with prolonged disease free survival and/or overall survival [10–12, 18–27]. Hence, the ATA thyroid guidelines recommend that as unresected ATC is almost certainly lethal, surgery should be performed when technically possible and if not likely to cause unacceptable morbidity [13]. According to TNM staging [28], all ATCs are stage IV. Stage IVA lesions are intrathyroidal, stage IVB involves gross extrathyroidal extension, and stage IVC disease includes distant metastasis. Stage IVA tumours are resectable; however, stage IVB tumours may be considered either resectable or unresectable. This decision will depend largely on local technical expertise of the surgeon and so in reality this distinction can be very variable. The ATA guidelines for ATC recommend that the aim of surgery is for gross clearance rather than tumour debulking. Using Edge et al.'s residual tumour classification as shown in Table 2 [28], ATA guidelines recommend surgery when a result of R0/R1 can be achieved; however, incomplete resection or tumour debulking (R2) is not recommended. This is contradictory to Haigh et al.'s [12] findings who noted aggressive surgery to be worthwhile in selective cases when combined with chemotherapy and/or radiotherapy even if some macroscopic disease has to be left behind to preserve organ function. This would reflect our own findings, as all surgically treated patients were noted to have residual macroscopic disease in our series (R2); yet a prolonged overall survival was observed compared to nonsurgically treated patients. Indeed, our one long-term survivor (>8 years+ at time of follow-up) also had tumour debulking rather than macroscopic clearance prior to radiotherapy:

*Treatment regimens that include surgical resection are associated with prolonged disease free survival and/or overall survival. The aim of surgery is for gross clearance rather than tumour debulking. Occasionally it may be necessary to leave residual macroscopic disease in order to preserve organ function.*

### 4.3. Radiotherapy

The ATA guidelines recommend that definitive radiotherapy should be offered after complete or near complete surgical resection (R0/R1) in patients with a good performance status with no metastatic disease. This recommendation largely comes from the Surveillance, Epidemiology, and End Results (SEER) database of 516 patients from which Kebebew et al. found that this combination of treatment was associated with lower cause-specific mortality on multivariate analysis [8]. Studies also suggest that high dose radiotherapy can also be of benefit for patients who have only had an R2 resection, have unresectable disease, or have limited metastatic disease [29] in order to improve survival by controlling local symptoms, plus by potentially reducing tumour size, hence allowing subsequent surgery.

The dose of radiotherapy appears to be of importance with several studies demonstrating a clear improvement in survival of patients given higher doses of radiotherapy. For example, Wang et al. [30] reported a median overall survival of 11.1 months in patients treated with radical radiotherapy (>40 Gy) compared to only 3.2 months in patients treated with a palliative dose (<40 Gy) (*P* < 0.001), a result reflected in several other studies [10, 11, 23, 30]. High dose radiation was considered to be 40 Gy or greater in most studies. Performance status may play a part in these results, but the message still remains that high dose radiotherapy is optimal if performance status allows.

Intensity-modulated radiation therapy (IMRT) is an advanced mode of high-precision radiotherapy that allows the radiation dose to conform more precisely to the 3D shape of the tumour, hence allowing higher radiation doses to be focused on complex tumour shapes whilst sparing surrounding normal structures. This allows higher and more effective radiation doses to be delivered to tumours with fewer side effects. Although IMRT has been suggested to improve outcome with reduced side effects in other head and neck and differentiated thyroid cancers [31–34], there is currently a lack of clear evidence for this with ATC. However, due to the favourable results for non-ATC patients, when available, IMRT may be a preferred option for ATC patients compared to conventional radiotherapy [35]:

*Definitive radiotherapy should be offered ideally after complete or near complete surgical resection (R0/R1) in patients with a good performance status with no metastatic disease. Higher doses (>40 Gy) and IMRT are preferable to improve outcome and reduce toxicity.*

### 4.4. Asphyxiation

Radiotherapy given solely to prevent death from asphyxiation may not be worthwhile. Certainly, in our series in which the median dose given was 30 Gy, radiotherapy to prevent death from asphyxiation was futile. Surgery plus higher dose radiation may be required. Additionally, strategies using hyperfractionated concurrent chemoradiotherapy seem to be more effective in local control than conventional fractionation or radiotherapy alone [36, 37]. Randomised studies incorporating QOL assessment are needed to confirm these findings. The ATA thyroid guidelines recognise the controversy regarding tracheostomy placement for airway preservation [8, 13, 19, 38–40], stating that tracheostomy or stent placement is best avoided unless there is impending airway compromise. However, this is noted to only be of temporary benefit as these patients are likely to have an extremely poor prognosis. Attempt at asphyxiation prevention via surgical excision is not recommended unless as part of tracheostomy insertion (isthmusectomy or debulking of pretracheal tumour for access) rather than as a definitive procedure on its own [13]:

*Low dose radiotherapy alone to prevent death from asphyxiation may be futile. Tracheostomy or stent placement is best avoided unless there is impending airway compromise, and is likely to be of only temporary benefit. Surgical excision is only recommended if part of a tracheostomy insertion.*

### 4.5. Chemotherapy

If patient performance status allows, most studies suggest that multimodality treatment regimens which include chemotherapy are of benefit to produce the longest median survival [10, 12, 18, 21, 41–43]. There is currently no general consensus as to what chemotherapy regimens are best though several studies have compared different agents [5, 10, 12, 21, 36, 44–46]. The ATA guidelines summarise the trials by recommending some combination of a taxane (paclitaxel or docetaxel) and/or anthracyclines (doxorubicin) and/or platin (cisplatin or carboplatin). These are recommended as part of multimodal therapy [13]. If facilities allow for the quick setup and initiation of concurrent treatment either post-op or in the inoperable setting, concurrent chemoradiotherapy is thought to produce the greatest survival advantage [10, 12, 45–47]. However, side effects and the possible deterioration in quality of life whilst undergoing toxic regimens must be considered when embarking on multimodal therapy. Hence, importance should be placed on the patients active involvement in the decision making process. Future research which includes less toxic chemotherapeutic regimens incorporating QOL assessment in clinical trial settings is urgently needed:

*Multimodality regimens which include chemotherapy improve median survival outcomes. Due to QOL issues whilst undertaking toxic regimens, importance is placed on the involvement of patients in the decision making process, balancing survival benefit versus possible side effects.*

### 4.6. Biological Agents/Trials

Although the literature suggests that multimodal therapy improves survival of ATC patients, outcomes are still poor. There is even less evidence to substantiate whether these toxic regimens are actually of benefit in terms of quality of life and survival of patients specifically with advanced metastatic disease [13]. Hence, research into novel agents that improve outcomes whilst limiting toxicity is ongoing and vital. A range of molecular targeting agents that have been tested in small clinical trials of ATC (summarised in Table 3) might represent viable therapeutic options with a promising disease control rate ranging from 33% to 75% [48]. Another recent phase II/III study (FACT trial) using carboplatin, paclitaxel, and fosbretabulin (CA4P) versus carboplatin and paclitaxel looks promising as it reported a 1-year survival of 33.3% versus 7.7%, respectively [49]. The trial was closed early due to low accrual rate. A larger sample size trial is needed to confirm statistically significant difference in survival. As per ATA guidelines, patients with advanced or metastatic ATC wishing an aggressive approach should be encouraged to enter clinical trials investigating novel biological agents:

*Research into novel agents that improve outcomes whilst limiting toxicity are ongoing and vital. Patients with advanced or metastatic ATC wishing an aggressive approach, should be encouraged to enter clinical trials investigating novel biological and molecular targeting agents.*

## 5. Conclusion

Results from our case series of 20 patients with ATC are poor with median survival at 59 days and one-year survival at only 5%. The addition of chemotherapy to our treatment regime led to an observed improvement in survival. In order to improve outcomes of ATC, a multimodality approach incorporating surgery, radiotherapy, and chemotherapy needs to be adopted when patient performance status allows. Chemotherapy appears to increase survival; however, toxic regimens can be poorly tolerated. Research into novel molecular targeting agents is urgently required.

## Figures and Tables

**Table 1:** Number of patients receiving each treatment modality and percentage dying from asphyxiation.

Treatment	Number of patients	Median survival (days)	Range (days)	Death from asphyxiation (%)		
				Yes	No	Unknown
None	1	32	n/a	0 (0)	1 (100)	—
Surgery	3	38	32–238	1 (33.3)	2 (66.7)	—
Radiotherapy	6	58.5	42–206	2 (33.3)	3 (50)	1 (16.7)
Chemotherapy	3	137	41–175	1 (33.3)	2 (66.7)	—
Surgery and radiotherapy	4	176	41–long-term survival (next highest 210)	1 (25)	2 (75)	(1 patient alive)
Chemotherapy and radiotherapy	3	220	52–350	3 (100)	0 (0)	—

**Table 2:** Residual tumour classification from edge et al.; AJCC cancer staging manual [28].

Residual tumour classification	Description
R0	No residual tumour
R1	Microscopic residual tumour
R2	Macroscopic residual tumour
RX	Presence of residual tumour cannot be assessed

**Table 3:** Trials using molecular targeting agents in ATC.

Study	Year	Phase	Setting	Number of patients	Agent	Mechanism of action	Overall survival
NCT01240590 (ClinicalTrials.gov)	2010 ongoing	I/II	Recurrent, metastatic, and inoperable	46 (planned)	Crolibulin plus cisplatin versus cisplatin alone	Microtubulin inhibitor	Awaiting
Savvides et al. [50]	2013	II	Advanced pretreated ATC	20	Sorafenib alone	Multikinase inhibitor with activity against Raf, VEGF, PDGFR, Ret, and c-kit	20% (1 year survival)
Sosa et al. [49]	2012	II/III	Advanced pretreated ATC	80	Fosbretabulin (CA4P) or placebo in combination with carboplatin/paclitaxel	VDA/tubulin-binding compound	33% (1 year compared to 7% in control arm)
Ha et al. [51]	2010	II	Advanced pretreated ATC	11	Imatinib alone	TKI acting upon Bcr-ABL and PDGF	45% (6 months)

ATC: anaplastic thyroid carcinoma; VEGF: vascular endothelial growth factor; PDGFR: platelet derived growth factor receptor: CA4P: Combretastatin A-4 phosphate; VDA: vascular disrupting agent; TKI: tyrosine kinase inhibitor.
